# Myositis-specific and myositis-associated autoantibodies in idiopathic inflammatory myopathies: An EASI survey on current clinical and laboratory practices

**DOI:** 10.1016/j.jtauto.2026.100398

**Published:** 2026-09-01

**Authors:** W.H.M. Vroemen, M.A. Dragon-Durey, Y. Allenbach, T. Berki, S. Čučnik, M. Infantino, E. Neves, E. Nagy, N. Pascu, D. Patel, M. Probst, L. Taylor, J.G.M.C. Damoiseaux, C. Bonroy

**Affiliations:** a Central Diagnostic Laboratory, Maastricht University Medical Centre, Maastricht, the Netherlands; b Université Paris Cité, Laboratory of Immunology, Hôpital Européen Georges-Pompidou, Assistance Publique Hopitaux de Paris (APHP), Paris, France; c Sorbonne Université, INSERM, Department of Internal Medicine, AP-HP, Hôpital Pitié-Salpêtrière, Paris, France; d Department of Immunology and Biotechnology, Medical School, University of Pécs, Pécs, Hungary; e Department of Rheumatology, University Medical Centre Ljubljana, Ljubljana, Slovenia; f Faculty of Pharmacy, University of Ljubljana, Ljubljana, Slovenia; g Immunology and Allergology Laboratory, S. Giovanni di Dio Hospital, Florence, Italy; h Department of Immunology, Hospital de Santo António, Centro Hospitalar Universitário do Porto, Porto, 4099-001, Portugal; i Department of Laboratory Medicine, Semmelweis University, Budapest, Hungary; j Department of Immunology- Autoimmunity, Central Laboratory, Regina Maria, CMU, Bucharest, Romania; k UK NEQAS Immunology, Immunochemistry & Allergy (IIA), Sheffield Teaching Hospitals, UK; l EUROIMMUN Medizinische Labordiagnostika AG, Lübeck, Germany; m Department of Immunology, Black Country Pathology Services, West Midlands, Wolverhampton, UK; n Department of Laboratory Medicine, Ghent University Hospital, Ghent, Belgium; o Department of Diagnostic Science, Ghent University, Ghent, Belgium

**Keywords:** Myositis, Diagnostics, Harmonization, Questionnaire

## Abstract

**Objectives:**

Detection of myositis-specific and myositis associated autoantibodies (MSA/MAA) aids in classification, diagnosis, and risk stratification of idiopathic inflammatory myopathies (IIM). To identify opportunities for harmonization, we assessed laboratory and clinical variability in IIM serological testing.

**Methods:**

The European Autoimmunity Standardisation Initiative (EASI) developed questionnaires targeting laboratory medicine specialists and clinicians addressing test initiation, testing methods and algorithms, reporting and interpretation. This was distributed through two external quality assessment (EQA) providers (UK NEQAS and IfQ Lübeck) to laboratory medicine specialists and subsequently to clinicians involved in IIM diagnostics.

**Results:**

In total 100 laboratory medicine specialists and 56 clinicians responded. Clinicians prefer to restrict requests for MSA/MAA to clinicians specialized in IIM and 97% of them agree with the indications that ask for MSA/MAA analysis as proposed during the 256th European NeuroMuscular Center (ENMC) workshop. Analysis was almost exclusively performed by commercially available assays (99%). Most laboratories test the ENMC recommended specificities (≥95%), with exception of HMGCR (22%). Both ISO15189 accreditation coverage (39-68%) and antigen-specific verification rates (33-64%) were suboptimal. Implementation of IQC was poor. Primary screening for MSA/MAA using HEp-2 indirect immunofluorescence was considered inappropriate by the majority of laboratory medicine specialists (59%) and clinicians (69%). Result reporting is most often done (semi-)quantitatively (75%) as also preferred by clinicians (91%).

**Conclusions:**

Considerable variability exists in MSA/MAA test initiation, testing methods and algorithms, reporting and interpretation. Introducing clinical- and laboratory-specific recommendations may contribute to harmonizing the interpretation of routine diagnostics for IIM.

## 1 Introduction

Idiopathic inflammatory myopathies (IIMs), commonly known as myositis, encompass a heterogeneous group of rare immune-mediated disorders primarily affecting skeletal muscle, with frequent extramuscular involvement of the skin, lungs, joints, heart, and other organs [[1], [2], [3]]. The clinical spectrum includes dermatomyositis (DM), polymyositis, immune-mediated necrotizing myopathy (IMNM), antisynthetase syndrome, inclusion body myositis, and overlap myositis, each associated with distinct histopathological, prognostic, and therapeutic characteristics [4,5]. Detection of myositis-specific autoantibodies (MSAs) and myositis-associated autoantibodies (MAAs) has become essential for accurate IIM classification, diagnosis, and risk stratification [[6], [7], [8]]. MSA are (highly) specific and typically mutually exclusive, detectable in 50–80% of cases, and strongly linked to clinical phenotypes, disease course, and complications such as interstitial lung disease, malignancy, or treatment refractoriness. MAAs, while less specific, often signal overlap syndromes and influence extra muscular manifestations [6].

MSAs and MAAs are increasingly incorporated into classification criteria and diagnostic algorithms. The 2017 EULAR/ACR criteria include only one autoantibody (anti-Jo1), yet it was given the highest weighting among all variables. Emerging evidence now supports expanding the panel of included autoantibodies for improved phenotypic accuracy [7,9]. Subtype-specific criteria (e.g. for IMNM and DM) position MSAs as key diagnostic elements, potentially obviating biopsy in seropositive patients [10,11]. Despite these advances, reliable MSA/MAA detection poses ongoing challenges. While immunoprecipitation is considered the gold standard, it involves a variety of conceptually distinct immunoassays, it is labour-intensive and mostly restricted to research laboratories related to expert/referral centres [12]. Routine diagnostics generally rely on commercial immunoassays (e.g. dot immunoassay (DIA), line immunoassay (LIA), enzyme-linked immuno-sorbent assay, chemiluminescence immunoassay), which improve accessibility and throughput, but show variable analytical and clinical performance characteristics [6,7,[12], [13], [14], [15]].

In particular, the commercial multiplex DIA/LIA are predominantly used for routine MSA/MAA detection in Europe [7,[15], [16], [17]]. These assays have the advantage that they can generate parallel data on a panel of antibodies in one single analysis, including also rare autoantibodies. Laboratory challenges related to the application of these DIA/LIA are quality control and assay verification of all antibodies included [7,16]. Moreover, these assays may differ in the number of antigens they include (thus the composition of the ‘myositis panel’) and the specific type of antigens used (e.g. single or multiple subunits, recombinant versus native), further contributing to variability in test results. Several authors have also suggested the adaption of manufacturer-defined cut-offs to improve diagnostic performance [6,16,18,19]. Recently, a study with 15 participating laboratories evaluating inter-laboratory variability in both analysis and reporting documented notable inconsistencies in how different users of the same assay interpret and report their results [15]. Laboratories may also differ in their test algorithm (e.g. positioning of HEp-2 indirect immunofluorescence (IIF)) and confirmation strategy (if present) [15]. In an effort to address some of these points of variability, a collaborative European NeuroMuscular Center (ENMC)/European Autoimmunity Standardisation Initiative (EASI) workshop in 2021 (published 2022) proposed consensus recommendations regarding the optimal testing strategy for patients suspected of IIM, and the format for reporting results to the clinician in order to enable optimal interpretation [20]. In this workshop a consensus proposal was also launched on the indications that may ask for a MSA/MAA test [20].

In light of ongoing harmonization initiatives, including the 2022 ENMC consensus recommendations, the EASI conducted international surveys targeting laboratory medicine specialists and clinicians to objectively document current practices and the extent of variation. This parallel approach also enabled the evaluation of alignment between laboratory services and expectations from clinical care, including the interpretation of test results. The ultimate goal is to promote harmonization of autoantibody diagnostics, achieve consensus on assay application and reporting, and thereby improve interpretation of routine myositis diagnostics across Europe. The present study reports the key findings from this EASI questionnaire and discusses them in the context of existing consensus efforts.

## 2 Materials and methods

On behalf of the EASI, two anonymous, web-based questionnaires were developed in Google Forms (drafted by MD, CB and JD) to address various aspects of multiplex testing in the context of IIM (see supplemental materials). The first survey was intended for laboratory medicine specialists and focused on the used test methodology including the testing algorithm, test interpretation/reporting and clinical context of the requests and quality assurance strategies. This questionnaire was distributed to laboratory medicine specialists via United Kingdom National External Quality Assessment Service (UK NEQAS) and/or Institut für Qualitätssicherung (IfQ) Lübeck based on the participation of medical laboratories in their external quality assessment (EQA) program for myositis antibodies on January 7th 2025 with a reminder sent on February 26th 2025. In total, 286 and 225 laboratories were addressed by UK NEQAS and IfQ Lübeck, respectively. Participants were asked to complete the survey only once if they received the request via both quality assurance organisations. The survey was available for completion over a 3-month period (January 2025 – March 2025). In parallel, a second survey intended for clinicians treating IIM patients was launched. This survey included questions focusing on test indications and ordering rules, laboratory interactions, knowledge about the test methodology and result interpretation. To reach the clinicians, laboratory medicine specialists receiving the first questionnaire were asked to invite 3–5 clinicians within their centre, preferably from different clinical disciplines, to complete the clinical survey. All participants (both laboratory experts and clinicians) were informed that for the survey the following MSA/MAA specificities are taken into account: Jo1, EJ, OJ, PL7, PL12, KS, ZO, HA, SRP, MDA5, TIF1ɣ, SAE, SAE1, SAE2, NXP2, Mi2, Mi2a, Mi2b, cN1A, HMGCR, PM/Scl, PM/Scl75, PM/Scl100, Ku. In addition, participants were informed on the ENMC consensus being a lead for some sections in the questionnaire [20]. Before data analysis survey responses were exported into Microsoft Excel (version 2311, Redmond, WA). Descriptive statistics were applied to summarize the quantitative responses and demographic data collected. Not all collected data from both questionnaires was included in this manuscript, but is available upon request.

## 3 Results

### 3.1 Geography and description of the participants

In total, 100 laboratory medicine specialists and 56 clinicians responded to the respective surveys. In addition to laboratories of European countries (n = 96), also laboratories from Australia (n = 1), Canada (n = 1), South Africa (n = 1) and New Zealand (n = 1) participated in the questionnaire (see Supplemental Table 1). Country information of participating clinicians was not collected.

The laboratory medicine specialists represented university hospital laboratories (62%, 62/100), non-university hospital laboratories (29%, 29/100), and private laboratories (9%, 9/100). Their main specialty was immunology (67%, 67/100), while few responders indicated to primarily have expertise in biochemistry/clinical pathology (9%, 9/100). Twenty-four laboratory medicine specialists (24%, 24/100) indicated polyvalent laboratory specialty. Most laboratory medicine specialists perform all MSA/MAA analyses in their own laboratory (63%, 63/100). Twenty-four laboratory medicine specialists (24%, 24/100) refer to another laboratory for some autoantibodies and 13 laboratory medicine specialists (13%, 13/100) sometimes refer in case of difficult cases or inconsistent findings. More than half of the laboratories (52%, 52/100) test between 26 and 100 samples per month, 36 (36%, 36/100) test 25 or less samples per month, while 11 (11%, 11/100) test more than 100 samples per month.

Most clinicians represented a university hospital (80%, 45/56). The remaining clinicians were from non-university hospitals (14%, 8/56) or had mixed public and private clinical activities (5%, 3/56). Their main specialty was rheumatology (45%, 25/56). The disciplines of the other clinicians were immunology (16%, 9/56), pulmonology (16%, 9/56), internal medicine (11%, 6/56), neurology (7%, 4/56) and dermatology (5%, 3/56). Most clinicians (45%, 25/56) had more than 15 years of experience in managing, treating, or diagnosing patients with (suspicion of) IIM. Twenty-one clinicians (38%) had between 5 and 15 years of experience, and the remainder (18%, 10/56) had less than 5 years of IIM experience. The majority of the 56 clinicians request maximum 5 (63%) or 6-10 (21%) MSA/MAA tests per month. Only 16% (9/56) of the clinicians ask more than 10 MSA/MAA tests per month.

### 3.2 Request limitations, test initiation and indications for requesting

Most laboratory medicine specialists agree (79%, 79/100) with the recent ENMC guidelines stating that MSA/MAA testing should not be requested by general practitioners (GPs), but patients should be referred to a clinical specialist depending on the dominant clinical manifestation [20]. This is also supported by the clinicians (86%, 48/56). Moreover, 75% of the clinicians (42/56) even want to limit the request to a selected set of disciplines. Nevertheless, the vast majority of laboratories (78%, 78/100) allow all clinical disciplines to request a myositis DIA/LIA analysis and 40 (40%, 40/100) even allow requests from GPs. Fifteen laboratories (15%, 15/100) only allow a selected set of clinical disciplines (predominantly rheumatology, immunology, internal medicine, pulmonology, dermatology and neurology). Seven laboratory medicine specialists (7%, 7/100) indicated that the specialty of the physician is not included when receiving an order for MSA/MAA testing.

Laboratory medicine specialists indicated that MSA/MAA testing is initiated for different reasons, but most are based on a direct request of the clinician (83%, 83/100) and/or the ANA pattern (79%, 79/100). Less commonly (48%, 48/100), laboratories initiate MSA/MAA testing based on clinical information independent of the original test request. Sixty-two laboratory medicine specialists (62%, 62/100) indicate they allow clinicians to request a myositis DIA/LIA independently from other laboratory tests. In contrast, 35 laboratories (35%) only allow a request in combination with HEp-2 IIF and 3 (3%) only perform the myositis DIA/LIA when HEp-2 IIF tested positive. Eighty-two percent of the clinicians (46/56) indicated that they are allowed to request a myositis DIA/LIA independently from other laboratory tests. However, 32 (57%, 32/56) indicated that they generally request it in combination with HEp-2 IIF (see also section 3.4.1 on the role of HEp-2 IIF). Once MSA/MAA testing is initiated most clinicians (71%, 40/56) prefer all available antigens to be tested for in the first request which is in line with the ENMC consensus [20], while the others (29%, 16/56) would limit the initial request to the relevant antibodies guided by the clinical context (e.g. only DM-MSA in presence of DM skin lesions).

Regarding request indications, almost all laboratory medicine specialists (97%, 97/100) agree with the clinical indications that ask for the initiation of MSA/MAA analysis as listed in the ENMC consensus [20]. From all the ENMC test indications the highest support (see Table 1) by the clinicians was given for the following isolated symptoms: interstitial lung disease of unknown cause (86%, 48/56), possible DM rash (84%, 47/56) and raised serum CK on repeated measurements (57%, 32/56). The support for the other isolated indications was ≤25% (range 4-25%). Higher support (≥93%) by the clinicians was given when there is also presence of other features of the myositis spectrum disorders.

**Table 1:** List of clinical symptoms in which the participating clinicians would test for MSA/MAA.

Clinical symptoms that may ask for a MSA/MAA test	In isolation n (%)	In presence of other features of myositis spectrum disorders
Raised serum CK on repeated measurements	32 (57%)	56 (100%)
Symptoms and signs of inflammatory arthritis/arthralgia	7 (13%)	54 (96%)
Unexplained fever	2 (4%)	53 (95%)
Possible dermatomyositis rash	47 (84%)	55 (98%)
Raynaud's syndrome	14 (25%)	55 (98%)
Cytoplasmic speckled^a^ identified on HEp-2 IIF	11 (20%)	52 (93%)
Interstitial lung disease of unknown cause	48 (86%)	55 (98%)
Clinical symptoms and signs suggestive of SLE	5 (9%)	
Clinical symptoms and signs suggestive of systemic sclerosis	22 (39%)	

a ANA patterns AC-19 and AC-20.

### 3.3 Test methodology and quality assurance

MSA/MAA analysis was almost exclusively (99%, 99/100) performed by commercial DIA/LIA from two companies; Euroimmun (LIA) and D-tek (DIA). Eighty-one laboratory medicine specialists (82%) reported using one DIA/LIA, 15 laboratory medicine specialists (15%) reported using two DIA/LIAs, and 3 laboratory medicine specialists (3%) reported using 3 or more DIA/LIAs for MSA/MAA testing. DIA/LIAs were predominantly obtained from a single commercial supplier (94%, 93/99), with only 6% of laboratory medicine specialists (6/99) sourcing DIA/LIAs from two suppliers. Of note, both suppliers, Euroimmun and D-tek, have several DIA/LIA with distinct antigen compositions in their portfolio. Ninety-two laboratories (92%, 92/100) indicated that they use an automated system for DIA/LIA incubation, while 8 laboratories (8%, 8/100) perform this manually. In addition, 96 laboratories (96%, 96/100) make use of a reader system for result analysis.

A majority of responding clinicians (57%, 32/56) reported awareness of the type of assay used for MSA/MAA testing. Nearly half of them (44%, 14/32) indicated to know the supplier of the MSA/MAA test employed in their own laboratory. In addition, almost all of them (91%, 29/32) believe that MSA/MAA test results obtained from different DIA/LIA suppliers are not of comparable quality.

According to the ENMC consensus recommendations the MSA tests should include the following specificities: HMGCR, SRP, TIF1 γ, NXP2, Mi2, MDA5, SAE, Jo1 and other aminoacyl tRNA synthetases (ARS; as far as available) [20]. Most laboratories adhere to the ENMC consensus recommendations by including SRP (99%, 99/100), TIF1γ (95%, 95/100), NXP2 (97%, 97/100), Mi-2 (100%, 100/100), MDA5 (97%, 97/100), SAE (97%, 97/100) and Jo1 (100%, 100/100) in their MSA testing repertoire. The sole exception of the ‘obligatory’ MSA was anti-HMGCR, which was only included in MSA testing by a minority of laboratory medicine specialists (22%, 22/100). The newer anti-ARS (Zo, HA, KS) are also tested only in a limited number of laboratories (24%, 24/100).

**Fig. 1:**
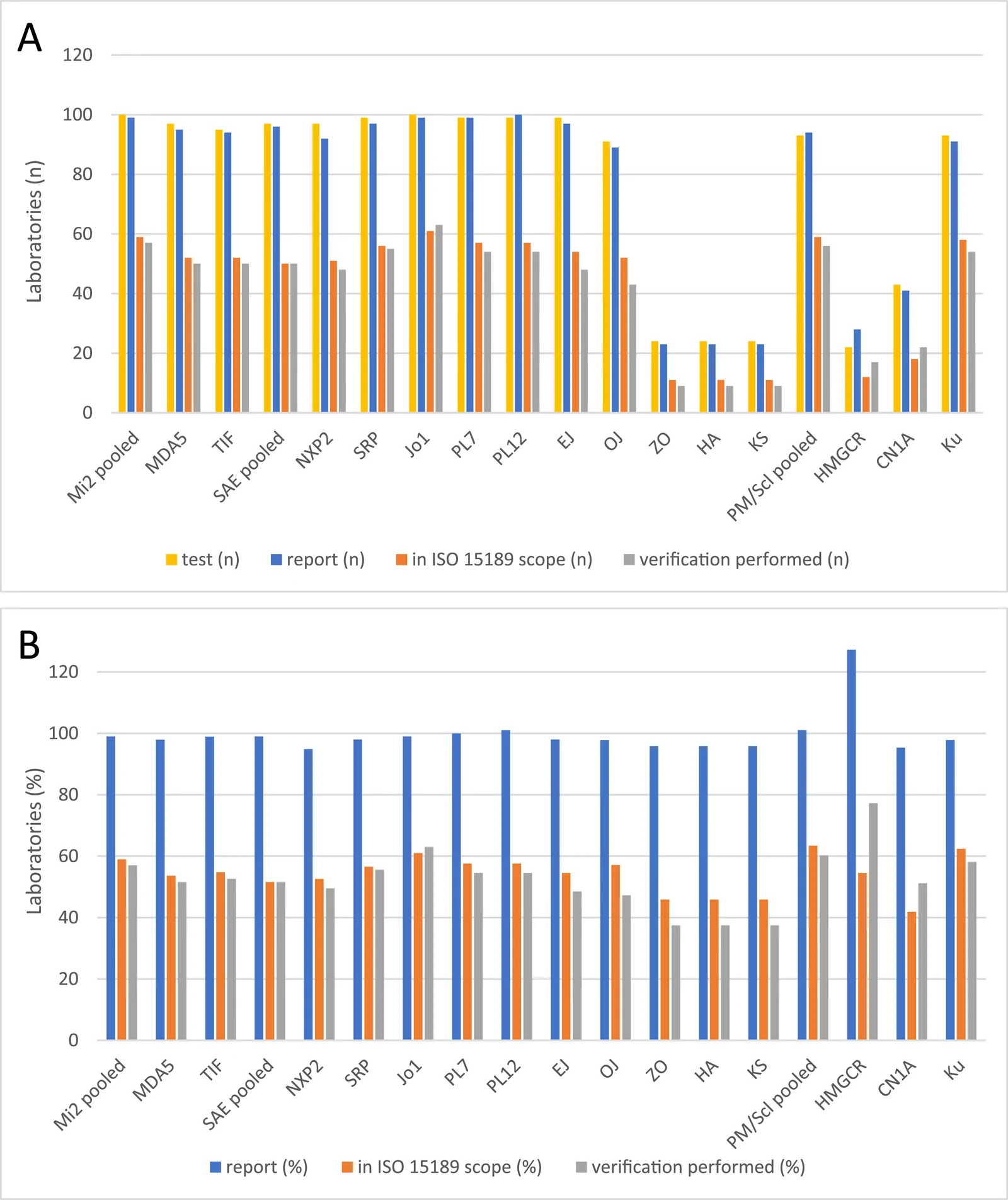
Number (A) and percentage (B) of laboratories, by antigen reactivity, that test for and report MSA/MAA results, include the corresponding test within their ISO 15189 scope, and perform antibody-specific verification. Percentages in panel B are calculated using, for each antigen reactivity, the number of laboratories that perform the respective test as the denominator. For HMGCR, more laboratories (n = 6) reported HMGCR results than indicated that they perform HMGCR testing. This discrepancy may be explained by laboratories referring HMGCR testing to other laboratories and subsequently reporting the results received. For PL12 and PM/Scl, the slight discrepancies resulting in reporting rates exceeding 100% may be attributable to errors in completing the questionnaire.

Most laboratory medicine specialists indicate to report the majority of the specificities they test for (see Fig. 1). Surprisingly, especially anti-HMGCR was indicated to be reported by responders more frequently than they were actually tested for. This is most likely explained by the fact that multiple laboratories refer for these autoantibodies to another laboratory. A substantial percentage of laboratories report MSA/MAA test results that are not verified (average verification rate 49%). Verification was most frequently performed for anti-Jo1 (64%) and least frequently for the more rare anti-ARS (33%). In line, only 39-68% of antigen specificities are covered within the laboratories' ISO 15189 accreditation scope.

Although the survey was distributed to laboratory medicine specialists via UK NEQAS and/or IfQ Lübeck based on their laboratories’ participation in external quality assessment (EQA) programs for myositis antibodies, not all respondents (96%, 96/100) reported participating in EQA for MSA/MAA testing. Seventy-seven percent of laboratories (74/96) participate in one EQA program, 16% (15/96) in 2 EQA programs and 2% (2/96) participate in more than 2 EQA programs. Additionally, 5% (5/96) of the laboratories provided an unclear answer.

**Fig. 2:**
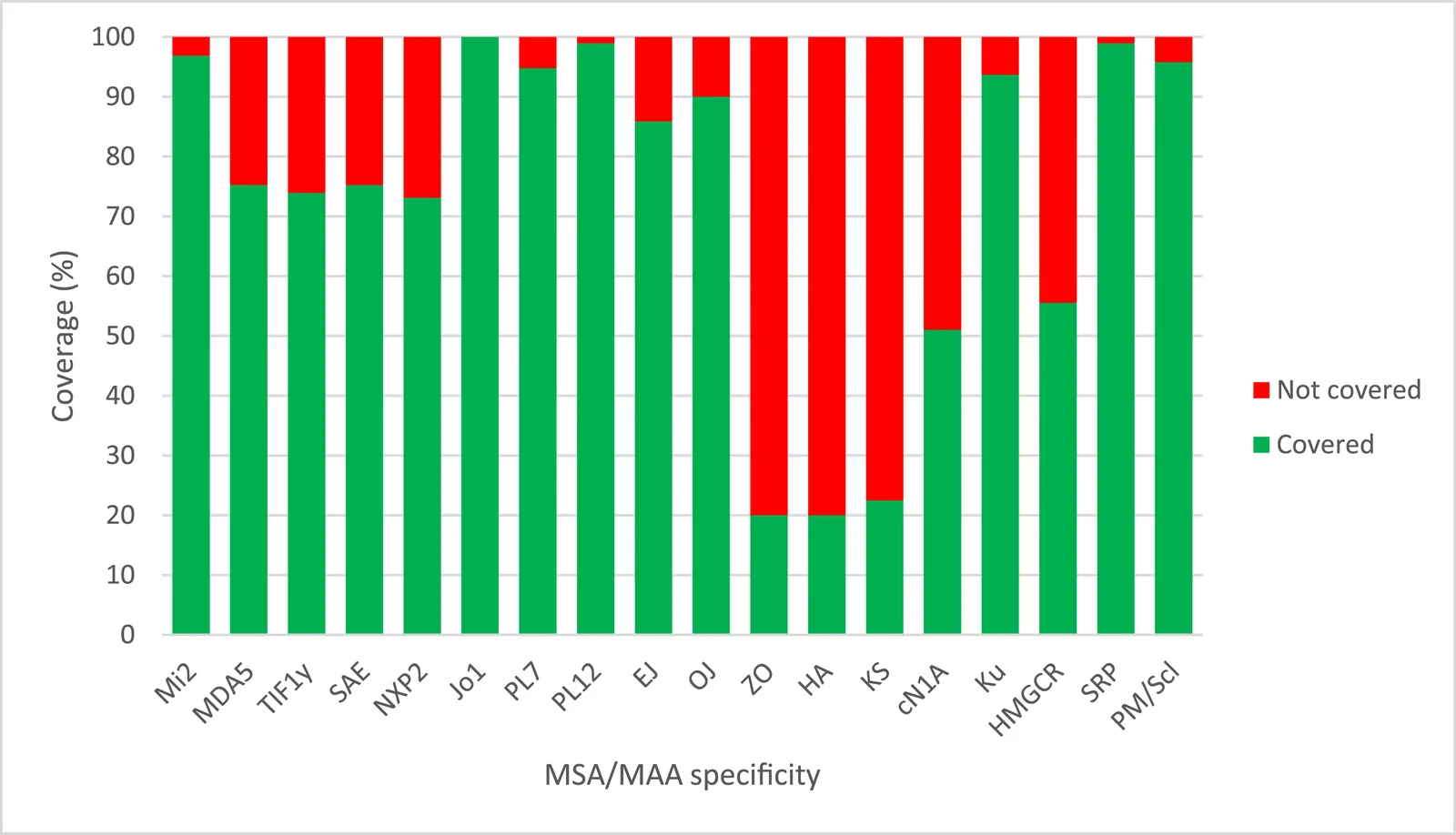
External quality assessment (EQA) coverage (%) per antigen specificity for those labs that indicate to participate in EQA (n = 96).

According to the laboratory medicine specialists that participate in EQA, the following antigen specificities were most frequently covered by their EQA programme during the last 3 years: Jo1 (100%), PL12 (99%), SRP (99%), Mi2 (97%), PM/Scl (96%), PL7 (95%) and Ku (94%). Of note, most EQA programs focus on the specificities that are tested by the majority of laboratories (Fig. 2). However, this implies that for some MSA a substantial proportion of laboratories that do test and report have no EQA coverage (ZO and HA (80%), KS (78%), CN1A (49%) and HMGCR (44%), partly due to the difficulty in obtaining suitable clinical samples in the volumes required.

**Fig. 3:**
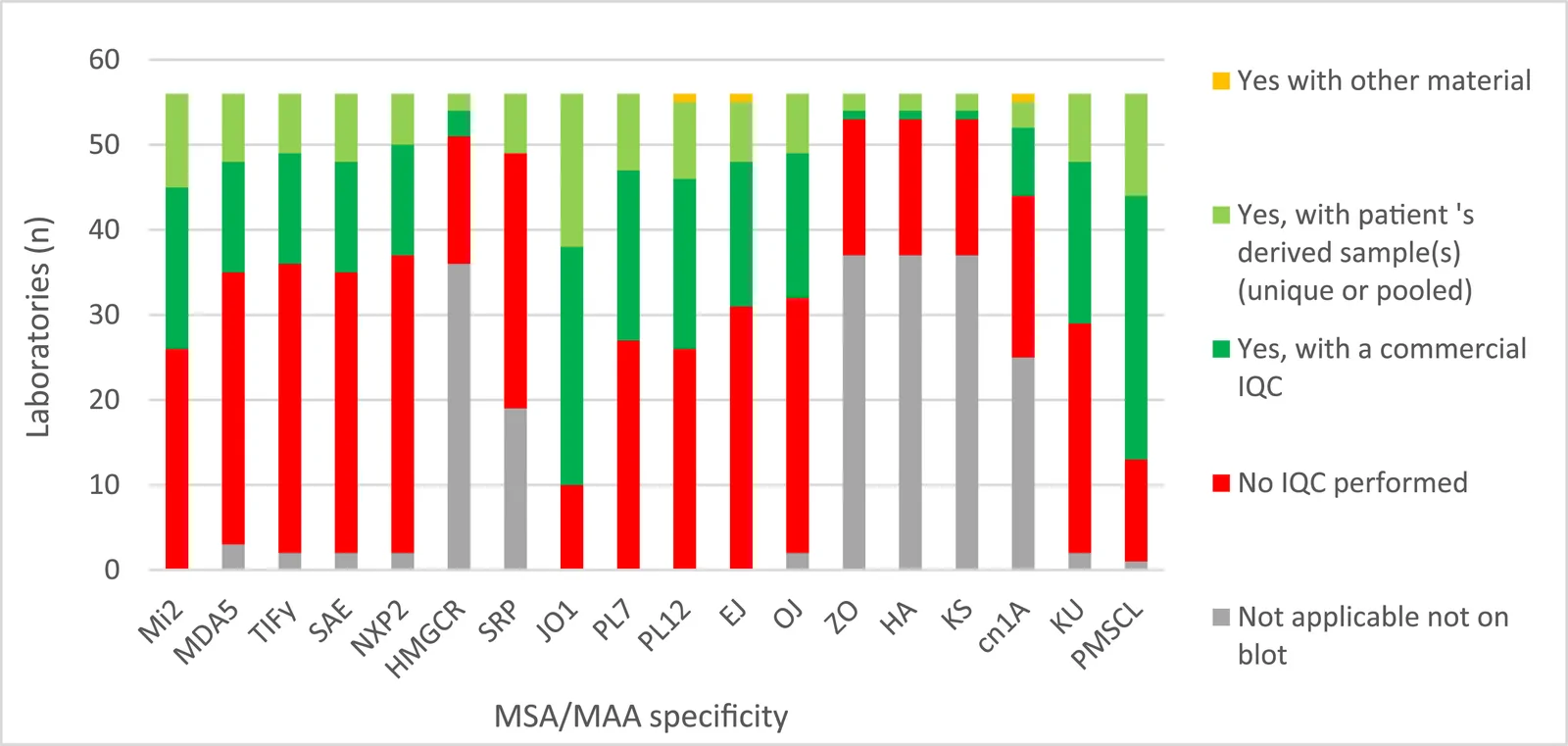
Internal quality control (IQC) coverage per antigen specificity for those labs that indicate to perform IQC (n = 56). Although not specifically addressed in the questionnaire, it is to be anticipated that commercial IQC is human derived. One lab uses other material for PL12, EJ and cN1A IQC purposes, but the questionnaire didn't allow further clarification about its origin.

Only 56 laboratories (56%) run an internal quality control (IQC) sample for at least one antigen specificity together with the patient samples. The average IQC coverage of the tested MSA is only 41% (range 16-82%) and the coverage per antigen specificity is depicted in Fig. 3. IQC is mostly performed using commercial (human-derived) IQC material (range 60-72%), with the exception of anti-SRP and the rare anti-ARS for which IQC material originates mostly from unique or pooled patient origin (100% and 67%, respectively). One lab uses other material for PL12, EJ and cN1A IQC purposes, but the questionnaire didn't allow further clarification about its origin. Additionally, most laboratories run the IQC during every run (57%, 32/56), some only after a reagent lot change (23%, 13/56), while 4 laboratories (7%, 4/56) run it at a fixed frequency independent of a reagent lot change and 7 laboratories (13%, 7/56) have another approach.

### 3.4 Testing algorithm

#### 3.4.1 Role of HEp-2 IIF testing

Although most MSA may give a positive HEp-2 IIF results [21,22], this is not evident with all specificities and the overall sensitivity is low [23]. For this reason the ENMC consensus recommendations consider HEp-2 IIF inappropriate for primary screening of MSA [20]. In line, the majority of laboratories (59%, 59/100) do not consider HEp-2 IIF appropriate for primary MSA/MAA screening. Clinicians seem also aware of this limitation, as only 31% (12/39) of the clinicians having an opinion on this topic state that HEp-2 IIF is a useful screening tool for MSA/MAA. The others consider HEp-2 IIF inappropriate (41%, 16/39) or only useful for some antigens (28% (11/39). Twenty-nine percent of clinicians (16/56) expressed to have no opinion and deferred to laboratory staff. Of note, in case of suspected IIM-overlap syndrome the ENMC recommends a direct assessment of MSA/MAA in combination with HEp-2 IIF, followed by an adequate algorithm for reflex as is generally accepted for the non-IIM systemic autoimmune rheumatic diseases [20].

Eighty-five laboratory medicine specialists (85%, 85/100) considered HEp-2 IIF patterns useful for supporting MSA/MAA findings. In contrast, only 46% (17/37) of clinicians use HEp-2 IIF results to interpret MSA/MAA findings. The others only use HEp-2 IIF for interpretation of a limited set of MSA/MAA specificities (19%, 7/37), while 35% did not use it at all (17/37). Notably, 34% of clinicians (19/56) referred to laboratory staff for an opinion on this issue.

#### 3.4.2 Confirmation and retesting

Most laboratories do not confirm positive results obtained in the first-line DIA/LIA with a second DIA/LIA and/or other methodology (not including HEp-2 IIF) for most antigen specificities (54-76%) due lack of availability of another assay. The only exception is anti-Jo1 which is confirmed with another antigen-specific immunoassay by 65 laboratories (66%). When confirmation is initiated it is more often done in specific situations (e.g. discrepancy between HEp-2 IIF and DIA/LIA, low antibody levels, inconsistent clinical findings, etc.) rather than upon specific request from the clinician. Confirmation is mostly performed by using a different myositis DIA/LIA (6%) or another immunoassay (FEIA, ELISA, CLIA; 59%) depending on availability.

Retesting for MSA/MAA when initial results are unexpectedly negative in a setting of high clinical suspicion is performed by the majority of clinicians (79%, 44/56). This may be warranted as per the consensus for patients on immune suppression during the initial evaluation [20]. Clinicians indicated that the moment of retesting is influenced by clinical changes (82%, 36/44), treatment resistance (45%, 20/44) and/or the time frame between samples (43%, 19/44).

When questioning how useful MSA/MAA monitoring is for patient follow-up, 46% of the clinicians (26/56) indicated that it is not useful. Other clinicians answered that retesting is useful to confirm the diagnosis (18%, 10/56) or to monitor treatment efficacy (20%, 11/56). Only 28% of laboratory medicine specialists consider monitoring not useful. More than the clinicians they do believe that retesting has an added value for confirmation of the diagnosis (43%). Regarding the added value for monitoring treatment efficacy laboratory medicine specialists have comparable opinions (17%) as clinicians.

Most laboratories (57%, 57/100) do not apply a minimal timeframe for MSA/MAA retesting, but when a timeframe is applied, most laboratories (49%, 21/43) use a 3-6 month time window. The retesting timeframe of the other 19 laboratories (44%) ranges from 3 to 365 days and 3 laboratories (7%) provided an unclear answer or leave this up to the clinicians.

### 3.5 Result reporting

The majority of laboratories report MSA/MAA results within 7 days (72%, 72/100) as per the ENMC consensus [20]. Twenty-two percent of the laboratories (22/100) report between 8 and 14 days and 6% (6/100) report their results later than 14 days. This aligns with the dominant clinical perspective preferring the results to be reported within 7 days (77%, 43/56). The other clinicians indicated that they accept it when results are reported within 8-14 days (23%, 13/56).

The way of reporting MSA/MAA test results is variable as well as the origin of the cut-off used to do the conversion to qualitative or semi-quantitative results (Table 2). The most common approach is semi-quantitative reporting (66%); quantitative reporting is only done by 9% (Table 2). Most laboratories that report in a qualitative or semi-quantitative manner use manufacturers determined cut-offs (76% and 81% respectively). This was both the case for Euroimmun users (93%, 76/82) and/or D-tek users (88%, 21/24). When asking the reporting preference (qualitative, semi-quantitative and/or quantitative) of clinicians most indicated they preferred (semi-)quantitative (91%, 51/56) above qualitative (9%, 5/56).

**Table 2:** Strategy of result reporting.

Type of reporting^a^	n (% of total of labs)
**Qualitative** (negative or positive)	**34 (34%)**
- based on in-house cut-off	8 (24%)
- based on manufacturer cut-off	26 (76%)
**Semi-quantitative** (in scores [e.g.-, +/−, +, ++] or in wording [e.g. weak, moderate, strong positive])	**66 (66%)**
- based on in-house cut-off	13 (19%)
- based on manufacturer cut-off	55 (81%)
**Quantitative** (as generated by the scanner)	**9 (9%)**
Other	2 (2%)

a Twelve laboratories use a combination of reporting strategies.

As it is known that DIA/LIA for detection of MSA/MAA may suffer from false (weak) positive results, we questioned both Euroimmun (n = 76) and/or D-tek users (n = 21) reporting qualitatively or semi-quantitatively what their threshold is for weak positive results. In this context it should be mentioned that according to the inserts of the kits for application of the EUROLineScan, equivalent results for the Euroimmun LIA are defined as 6-10U (8-14U by integrated camera photography for EUROBlotOne users), but should be reported as negative, while for the D-tek DIA equivalent results are defined as 5 – 10U without any instruction on how to report such results. Both companies do not refer to the term “weak positive”. Most Euroimmun users (68%) report 11-25U (15-35U for EUROBlotOne users) as weak positive (51/76), 13% (10/76) use 6-10U (8-14U for EUROBlotOne users) and 20% (15/76) use a different strategy (e.g. in-house cut-off values). Most D-tek users do not use the manufacturers range of 5-10U (38%, 8/21) for weak positive results, 14% laboratories (3/21) do not report weak positive results and 48% (10/21) use a different strategy (e.g. in-house cut-off values).

Since some MSA/MAA targets may be represented by two distinct antigens (Euroimmun: Mi2α and Mi2β, PM/Scl75 and PM/Scl100; D-tek: SAE1 and SAE2), we questioned how laboratories report these results. Most laboratories report both distinct antigens independently; 79% (65/82), 86% (81/94), 55% (21/38) for Mi2α and Mi2β, PM/Scl75 and PM/Scl100, and SAE1 and SAE2, respectively (Table 3). The mostly used alternative for Mi2 and PM/Scl is combining subunit results and reporting based on positivity of at least one subunit which is performed by 11% (9/82) and 11% (10/94) for Mi2 and PM/Scl, respectively. For SAE, the most used alternative is reporting based only on SAE1 (32%, 12/38).

**Table 3:** Result reporting of MSA/MAA represented by two distinct antigens.

	Number (% on total of labs testing subunits)
**Mi2 antibodies reporting**	**(n=82)**

Only Mi2α	0 (0%)
Only Mi2β	2 (2.4%)
Both Mi2α and Mi2β, independently	65 (79%)
Mi2 - based on positivity of at least one subunit	9 (11%)
Mi2 - based on positivity of both subunits	0 (0%)
Otherwise	6 (7.3%)

**PM/Scl antibodies reporting**	**(n=94)**

Only PM/Scl75	0 (0%)
Only PM/Scl100	0 (0%)
Both PM/Scl75 and PM/Scl100, independently	81 (86%)
PM/Scl - based on positivity of at least one subunit	10 (11%)
PM/Scl - based on positivity of both subunits	0 (0%)
Otherwise	3 (3.2%)

**SAE antibodies reporting**	**(n=38)**

Only SAE1	12 (32%)
Only SAE2	0 (0%)
Both SAE1 and SAE2, independently	21 (55%)
SAE - based on positivity of at least one subunit	3 (7.9%)
SAE - based on positivity of both subunits	0 (0%)
Otherwise	2 (5.3%)

Multiple reactivities are also differentially reported. Sixty-seven percent (67/100) of laboratories report all MSA results as they are, with the majority of them also adding a comment (45/67). Alternative strategies include reporting all (18%) or some (16%) of the weak positive MSA as negative. Twenty percent (20/100) use a confirmation assay to further elaborate such sample.

### 3.6 Result interpretation

When asking the clinicians if they considered weak positive MSA/MAA results clinically relevant, most (57%, 32/56) indicated that this is dependent on the pre-test probability and/or antibody specificity. Another large proportion of clinicians (27%, 15/56) will discuss such case with a laboratory medicine specialist to decide on the clinical relevance. Others (9%, 5/56) were of opinion that the clinical relevance of weak positive results is independent of clinical information but dependent on the antibody specificity, while 2% (1/56) believed a weak positive result is never relevant.

A substantial part of laboratory medicine specialists and clinicians believe there is no added value subtyping for Mi2, PM/Scl and SAE (laboratory medicine specialists 21-26%, clinicians 13-35%), or they indicated that they don't know if it is of added value (laboratory medicine specialists 28-47%, clinicians 37-71%) (Table 4). A substantial part of laboratory medicine specialists also indicated for Mi2 and PM/Scl that double positivity is more relevant than single reactivity (28% and 23%, respectively). A minority of laboratory medicine specialists and clinicians are of the opinion that for Mi2 and PM/Scl one of the distinct antigens is superior to the other, and if they indicate superiority, Mi2b (12%) and PM/Scl100 (16%) are believed to be more relevant. SAE1 superiority is indicated by 21% of laboratory medicine specialists, most clinicians do not know.

**Table 4:** Clinical relevance of subtyping on Mi2, PM/Scl and SAE antibodies.

	Labs (% on total of labs testing subunits)	Clinicians (% on total that state that labs report subunits)
**Mi2 antibodies clinical relevance**	**(n=82)**	**(n=45)**

Mi2b > Mi2a	10 (12%)	1 (2,2%)
Mi2a > Mi2b	1 (1,2%)	5 (11%)
Mi2b = Mi2a, no relevance of subtyping^a^	21 (26%)	14 (31%)
Double positivity more relevant than single positivity	23 (28%)	4 (8,8%)
Don't know	27 (33%)	21 (47%)

**PM/Scl antibodies clinical relevance**	**(n=94)**	**(n=54)**

PM/Scl-100 > PM/Scl-75	15 (16%)	4 (7,4%)
PM/Scl-75 > PM/Scl-100	1 (1,1%)	4 (7,4%)
PM/Scl-100 = PM/Scl-75, no relevance of subtyping^a^	30 (32%)	19 (35%)
Double positivity more relevant than single positivity	22 (23%)	7 (13%)
Don't know	26 (28%)	20 (37%)

**SAE antibodies clinical relevance**	**(n=38)**	**(n=48)**

SAE1 > SAE2	8 (21%)	3 (6,3%)
SAE2 > SAE1	0 (0%)	0 (0%)
SAE1 = SAE2, no relevance of subtyping^a^	8 (21%)	6 (13%)
Double positivity more relevant than single positivity	4 (11%)	5 (10%)
Don't know	18 (47%)	34 (71%)

a Combination of 2 result options: ’I think both reactivities are equally relevant‘ and ‘I think that there is no added value of subdifferentiating’.

With respect to the clinical relevance of multi-reactivity, most clinicians (55%, 31/56) believe this depends on the pre-test probability and/or antibody levels. Twenty-seven percent of clinicians (19/56) would discuss the case with the laboratory specialist to decide on the clinical relevance. Others (5%, 3/56) believe it solely depends on the antibody levels and 5% (3/56) believe multi-reactivity is never clinically relevant.

Another important result interpretation aspect is the management of discordant results in case a confirmation assay is performed. Most laboratories (54%, 37/68) report all the individual results of the different tests and include a comment, others (34%, 23/68) integrate the individual results and report one combined result interpretation, while the minority (12%, 8/68) report all individual test results of the different tests and let the clinician conclude.

Overall, clinicians (strongly) agree that the impact of MSA/MAA testing influence their diagnostics performance (91%, 51/56), the information the clinician provides to their patient on prognosis (86%, 48/56), additional investigations (88%, 49/56) and the recommended treatment (89%, 50/56).

## 4 Conclusion

We report the findings of two simultaneous international surveys that evaluated the current routine practices of MSA/MAA testing in IIM. The primary aims were to document existing testing practices and to assess the extent of variation between laboratories and clinicians, particularly in the context of ongoing harmonization efforts, including the 2022 ENMC consensus recommendations. By targeting both laboratory medicine specialists and clinicians, the surveys enabled a direct evaluation of the alignment between laboratory services and clinical practice. Specifically, the surveys examined key aspects of testing, including clinical indications and initiation of requests, request limitations, test methodologies, quality assurance measures, testing algorithms, as well as reporting and interpretation practices. Our findings indicate that most laboratory medicine specialists and clinicians align with the 2022 ENMC consensus recommendations in some of the key aspects of MSA/MAA testing (e.g. the role of HEp-2 IIF as a screening test, the ‘all at once testing approach’ and test indications) [20]. There is also agreement with ENMC by laboratory medicine specialists as well as clinicians on the need for test limitations based on the clinical discipline of the requestor. Nevertheless, this seems not to be implemented in many laboratories, as the reinforcement of some limitations probably needs support of the healthcare policy makers. Despite these common practices, considerable variability in MSA/MAA testing was observed at multiple levels, potentially affecting interpretation of reported results. Significant deviations from the 2022 ENMC consensus warranting further discussion are mentioned below.

A notable deviation from the consensus is the absence of testing for anti-HMGCR in the MSA/MAA panel among most of the participants in this survey. However, the inclusion of this MSA remains diverse, as available DIA/LIA immunoassays mostly do not cover anti-HMGCR. Additionally, the available DIA do not include anti-HMGCR, whereas LIA variants including anti-HMGCR are not for sale in all countries. Given the low prevalence, along with the distinct clinical phenotype of IMNM (e.g., markedly elevated CK levels) and economical arguments, a clinically driven approach may also be appropriate.

From a laboratory accreditation perspective multiple quality assurance items need to be met. These include verification, participation in EQA and the use of IQC. This is particularly challenging for multiplex assays for rare diseases like IIM, where MSA/MAA themselves are extremely rare [17,24]. Our results indicated that a substantial proportion of laboratories performing and reporting these tests do not fulfil the ISO15189 requirements for these items. A possible solution for appropriate verification is proposed by the Dutch College of Medical Immunology and entails a national multicentre approach [25]. Another quality assurance point for improvement is IQC. Only approximately half of the laboratories include an IQC in each run while all kits from the two myositis DIA/LIA manufacturers contain a positive control. Although these controls only contain one or two autoantibody reactivities and cannot compensate for an optimal IQC reagent investigating the performance of all autoantibody specificities including in the assay, it should be run as a bare minimum to ensure the analysis is properly conducted.

Primary screening for MSA/MAA with HEp-2 IIF was considered inappropriate by the majority of laboratory medicine specialists and clinicians. Although the ENMC consensus concluded that the added value of HEp-2 IIF as the primary screening test is very limited in IIM, but should be performed upon suspicion of an IIM-overlap syndrome, there is still discussion on the added value of the pattern in support of a positive MSA/MAA finding [20,26]. Multiple research groups suggested that the positive predictive value of MSA/MAA in IIM can be improved if there is positive concordance with the HEp-2 IIF pattern [[27], [28], [29]]. However, the definition of a compatible pattern was not fully correct for some MSA/MAA [27] or broadly defined (e.g. for TRIM21/Ro52 a negative, fine speckled or cytoplasmic HEp-2 pattern were all considered compatible) and overrepresented and thereby increased concordance [28]. Others agreed that the variable concordance between LIA and immunoprecipitation, use of non-standardized assay thresholds, and the integration of HEp-2 IIF despite its limited sensitivity for certain MSA/MAA may collectively affect the reliability and generalizability of the reported diagnostic performance in routine clinical practice [30].

Approximately a quarter of the laboratory specialists and half of the clinicians indicated that MSA/MAA monitoring is not useful. To date, there is currently no apparent relation between individual MSA levels and disease activity that warrants repeat testing. However, Stone et al. found that anti-Jo1 antibody levels paralleled markers of myositis disease activity and were even not detectable in some patients during periods of disease inactivity [31]. Moreover, there is emerging evidence that anti-HMGCR antibodies may be useful for monitoring the clinical course in individual patients [32]. Also, Benveniste et al. revealed that monitoring anti-SRP levels correlated with surrogate disease activity markers [33]. Nevertheless, the British Society of Rheumatology guidelines for managing IIM as well as the ENMC consensus do not recommend measuring autoantibody levels to monitor disease activity based on insufficient evidence, but this may be an area of interest for future research [20,34].

Detection of autoantibodies directed to multiple isoforms of Mi2, PM/Scl and SAE is performed although it is not available for all MSA by all manufacturers. In addition, if subtyping is not performed, the package insert does not clearly specify which isoform is actually detected by the DIA/LIA. The reasons for targeting variants on these particular antigens are not clear and publications on the added clinical value are limited. In our survey, approximately half of the laboratory medicine specialists and clinicians indicated they don't know if it is of added value, while still a substantial part believes double positivity is more relevant than single positivity or that one isoform is superior to the other. More extensive studies are needed to evaluate if subtyping can improve diagnostic accuracy or provide clinically meaningful disease stratification [7].

A substantial proportion of laboratory medicine specialists and clinicians are unsure about the interpretation of weak positive results, the clinical relevance of multi-reactivity, the value of subtyping for Mi2, PM/Scl and SAE, and how to manage discordant results. It is unknown if these respondents were located in the same institution. Continuous education of and collaboration between laboratory medicine specialists and clinicians is essential for result interpretation and includes understanding assay limitations, pre-test probability, and integrating serological data with clinical findings [17].

Our study has several limitations. First, the survey was distributed through EQA providers, which may have introduced a selection bias toward higher-performing laboratories, potentially leading to an overestimation of the implementation of quality measures. Second, clinicians were recruited through network sampling via laboratory medicine specialists, which may have resulted in network bias and limited representativeness. Third, the reasons for non-response were not investigated, which may affect the validity and generalizability of the findings.

Taken together, the findings of this study highlight persistent variability in MSA/MAA testing practices and interpretation, despite ongoing harmonization efforts. The parallel insights from laboratory medicine specialists and clinicians underscore the need for closer alignment between diagnostic services and clinical expectations, particularly regarding MSA/MAA testing including quality assurance, reporting, and interpretation of test results. In this context, recommendations from an international expert groups such as the International Myositis Assessment and Clinical Studies Group (IMACS), integrating both clinical and laboratory perspectives, could represent an important step toward reducing heterogeneity. Strengthening collaboration between disciplines and embedding such consensus guidance into routine practice may ultimately enhance the reliability and comparability of IIM diagnostics, thereby improving patient care.

## CRediT authorship contribution statement

**W.H.M. Vroemen:** Writing – review & editing, Writing – original draft, Visualization, Validation, Project administration, Formal analysis, Data curation. **M.A. Dragon-Durey:** Writing – review & editing, Methodology, Investigation, Data curation, Conceptualization. **Y. Allenbach:** Writing – review & editing, Methodology, Conceptualization. **T. Berki:** Writing – review & editing, Methodology, Conceptualization. **S. Čučnik:** Writing – review & editing, Methodology, Conceptualization. **M. Infantino:** Writing – review & editing, Methodology, Conceptualization. **E. Neves:** Writing – review & editing, Methodology, Conceptualization. **E. Nagy:** Writing – review & editing, Methodology, Conceptualization. **N. Pascu:** Writing – review & editing, Methodology, Conceptualization. **D. Patel:** Writing – review & editing, Methodology, Conceptualization. **M. Probst:** Writing – review & editing, Methodology, Conceptualization. **L. Taylor:** Writing – review & editing, Methodology, Conceptualization. **J.G.M.C. Damoiseaux:** Writing – review & editing, Supervision, Methodology, Conceptualization. **C. Bonroy:** Writing – review & editing, Writing – original draft, Visualization, Validation, Supervision, Methodology, Investigation, Formal analysis, Data curation, Conceptualization.

## Declaration of competing interest

The authors declare that they have no known competing financial interests or personal relationships that could have appeared to influence the work reported in this paper.

## Data Availability

Data will be made available on request.
