# Supplementary material for: Myositis-specific and myositis-associated autoantibodies in idiopathic inflammatory myopathies: An EASI survey on current clinical and laboratory practices
Source: J Transl Autoimmun. 2026 Sep 1;13:100398. doi: 10.1016/j.jtauto.2026.100398 (PMC13571532; doi:10.1016/j.jtauto.2026.100398)
Supplement: Supplementary file 1 [file mmc2.pdf]

EASI questionnaire on clinical interpretation of myositis specific/associated antibodies

Detection of myositis-specific autoantibodies (MSA) and myositis-associated autoantibodies (MAA) are increasingly requested in routine clinical settings.
Detection of these autoantibodies is most often performed by multiplex line or dot immuno-assays (myositis blot). Analysis, results interpretation and reporting appear to be heterogeneous and therefore prone to harmonization. A consensus on harmonization has been reached by the ENMC (Damoiseaux et al, NMD 2022) and this consensus is a lead for this questionnaire.

For this questionnaire the following MSA/MAA are taken into account: Jo1, EJ, OJ, PL7, PL12, KS, ZO, HA, SRP, MDA5, TIF1y, SAE1, SAE2, NXP2, Mi2a, Mi2b, cN1A, HMGCR, PM/Scl, PM/Scl75, PM/Scl100, Ku.

The aim of this questionnaire is to unravel the heterogeneity of test results and interpretation, with the ultimate goal to come to a consensus and alignment of the different aspects of the application of the myositis blot.

* Indicates required question

Section 1 : Characterization of the clinical activity

1. 1 . Your clinical activity is performed in: *

Mark only one oval.

- An university hospital
- A non-university hospital
- A private hospital
- I have mixed private and public
- activity Other

2. 2. What is your main specialty ? *

Mark only one oval.

- Immunology
- Rheumatology
- Internal Medicine
- Neurology
- Dermatology
- Pneumology
- Other

3. 3. Since how long are you managing/treating/diagnosing patients with (suspicion of) Inflammatory myopathy? *

Mark only one oval.

- <2 years
- 2-5 years
- 5-10 years
- 10-15 years
- >15 years

4. 4. How many tests do you ask per month for MSA/MAA? *

Mark only one oval.

- 0
- 1 to 5
- 6 to 10
- 11 to 20
- >20

Section 2: Prescription rules and test initiation

5. 5. In which patients would you typically test for MSA/MAA? *

In patients with:
Mark only one oval per row.

	Yes	No
Raised serum CPK on repeated samples in isolation	⬭	⬭
Raised serum CPK on repeated samples in presence of other features of myositis spectrum disorders	⬭	⬭
Symptoms and signs of inflammatory arthritis/arthralgia in isolation	⬭	⬭
Symptoms and signs of inflammatory arthritis/arthralgia in presence of other features of myositis spectrum disorders	⬭	⬭
Unexplained fever in isolation	⬭	⬭
Fever in presence of other features of myositis spectrum disorders	⬭	⬭

Possible DM rash in isolation	◯	◯
Possible DM rash in presence of other features of myositis spectrum disorders	◯	◯
Raynaud's syndrome in isolation	◯	◯
Raynaud's syndrome in presence of other features of myositis spectrum disorders	◯	◯
Cytoplasmic speckled (Anapatterns #AC19,20) identified on indirect immunofluorescence alone	◯	◯
Cytoplasmic speckled (Anapatterns #AC19,20) identified on indirect immunofluorescence in presence of other features of myositis spectrum disorders	◯	◯
Interstitial lung disease of unknown cause in isolation	◯	◯
Interstitial lung disease		

of unknown cause in presence of other features of myositis spectrum disorders	⬭	⬭
Clinical symptoms and signs suggestive for SLE	⬭	⬭
Clinical symptoms and signs suggestive of systemic sclerosis	⬭	⬭
Other clinical symptoms	⬭	⬭

6. 5a. In case your MSA/MAA request is guided by other clinical symptoms than listed above, please specify:

____

7. 6. In your opinion, which MSA/MAA reactivities must be tested at first request? *

Mark only one oval.

⬭ It is better to test all available MSA/MAA reactivities in the first request *Skip to question 9*

⬭ It is better not to test for all MSA/MAA at once, and limit the inital request to the relevant antibodies guided by the clinical context *Skip to question 8*

Section 2b: Prescription rules and test initiation

8. 7c. In your opinion, which reactivities must be tested according to the clinical context? *

Tick all that apply.

	ILD dominant symptoms	Dermatological dominant symptoms	Muscle dominant symptoms	Arthritis dominant symptoms	Cancer associated myositis	Reactivity not tested by the laboratory
Mi2/Mi2a/Mi2b	☐	☐	☐	☐	☐	☐
MDA5	☐	☐	☐	☐	☐	☐
TIF1y	☐	☐	☐	☐	☐	☐
SAE/SAE1/SAE2	☐	☐	☐	☐	☐	☐
NXP2	☐	☐	☐	☐	☐	☐
HMGCR	☐	☐	☐	☐	☐	☐
SRP	☐	☐	☐	☐	☐	☐
Jo1	☐	☐	☐	☐	☐	☐
PL7	☐	☐	☐	☐	☐	☐
PL12	☐	☐	☐	☐	☐	☐
EJ	☐	☐	☐	☐	☐	☐
OJ	☐	☐	☐	☐	☐	☐
Ks	☐	☐	☐	☐	☐	☐
Zo	☐	☐	☐	☐	☐	☐
Ha	☐	☐	☐	☐	☐	☐
cN1A	☐	☐	☐	☐	☐	☐
Ku	☐	☐	☐	☐	☐	☐
PM/Scl PM/Scl75 PM/Scl100	☐	☐	☐	☐	☐	☐

Section 2c: Prescription rules and test initiation

9. 7a. What disciplines do you think should be able to request MSA/MMA testing? *

Mark only one oval.

- ll disciplines (including GP)
- all disciplines (except GP)
- only a selection of disciplines

10. 7b. In case you think that only a selection of disciplines should be able to request MSA/MMA testing, please specify:

____

11 . 8. Is it possible for you to request a myositis blot independently from other laboratory tests? *

Mark only one oval.

- Yes, in general I request it alone
- Yes, but in general I request in combination with HEp-2 IFA
- No, only in combination with HEp-2 IFA
- No, the myositis blot will be performed only if HEp-2 IFA is tested positive
- Other:

Section 3: Interaction with the laboratory

12. 9. In which type of laboratory the majority of the MAA/MSA analysis that you request are analyzed? *

Mark only one oval.

- University hospital laboratory
- Non University hospital laboratory
- Private hospital laboratory
- Private laboratory
- I have no preference, the patients are choosing the laboratory

13. 10. In a 1 to 5 scale, how is your interaction with the laboratory performing the myositis blot to discuss the results? *

Mark only one oval.

	1	2	3	4	5	
No interaction	◯	◯	◯	◯	◯	Frequent interaction

14. 11. How confident are you of the MSA/MAA results delivered by your laboratory? *

Mark only one oval.

- ◯ Always confident
- ◯ Mostly confident
- ◯ Rarely confident
- ◯ My confidence varies depending on the autoantibody
- ◯ No opinion

15. 11 a. If your confidence depends on the autoantibody, please explain:

16. 12. How fast would you like to obtain your MSA/MAA test results? *

Mark only one oval.

- 1-3 days
- 3-7 days
- 8-14 days
- >14 days

Section 4: Knowledge about the methodology

17. 13. Do you know which type of assay (method and/or supplier) your local laboratory uses for identifying MSA/MAA? *

Mark only one oval.

- No *Skip to question 22*
- Yes *Skip to question 18*

Section 5: Details of the methodology

18. 14. Which type of assay does your local laboratory use for identifying myositis relevant autoantibodies? (select all that apply)*

Tick all that apply.

- Myositis line/dot blot
- Enzyme immunoasay/ELISA
- ALBIA (Laser bead)
- Immunoprecipitation
- Other

19. 14 a. If your laboratory use an other assay, please specify:

____

20. 15. Do you know the supplier of the MSA/MAA dot/line blot test your lab is using? *

Mark only one oval.

- Yes
- No

21. 16. Do you think the MSA/MAA results generated by tests from different suppliers generate the same quality of results?*

Mark only one oval.

- Yes
- No

Section 6: Results interpretation

22. 17. Do you know, without checking, how the results are reported (qualitative, quantitative, semi-quantitative)? *

Additional information:

- Qualitative reporting (positive/negative) =without providing further information on the level of the antibody signal

- Semi-quantitative reporting =with grading depending on the antibody level either in symbols (e.g. +, ++,+++) or in words (e.g. weak positive, moderate positive, strong positive, etc.)

Quantitative reporting= the antibody level is reported in numbers

Mark only one oval.

- ( ) Yes
- ( ) No

23. 18. What do you consider the best way to report the results? If you prefer a combination of reporting, please indicate multiple options. *

Tick all that apply.

- [ ] Qualitative
- [ ] Semi-quantitative
- [ ] Quantitative values

24. 19. Do you consider useful to confirm the myositis blot results by another assay when available? *

Mark only one oval.

- No
- Yes, but only for the low positive results
- Yes, for all positive results
- I have no opinion on this point, I refer to the laboratory staff

25. 19b. Do you use the results of ANA/HEp-2 for screening for MSA/MAA?

Mark only one oval.

- I have no opinion on this point, I refer to the laboratory staff
- No
- Yes
- Yes, for some antigens

26. 20. Do you use the results of ANA/HEp-2 to interpret the MSA/MAA results? *

Mark only one oval.

- I have no opinion on this point, I refer to the laboratory staff
- No
- Yes
- Yes, for some antigens

27. 20a. If yes, for some antigens, please justify your answer:

28. 21. Do you consider low positive MSA/MAA results clinically relevant? *

Mark only one oval.

- Never, independent on clinical information and antibody specificity
- Clinical relevance is independent of clinical information but dependent on the antibody specificity
- Clinical relevance is depending on the pre-test probability and/or antibody specificity
- No opinion, I refer to lab staff for interpretation
- I will discuss the case with the lab staff to decide on the clinical relevance
- Not applicable, I have only qualitative results (positive/negative, without information on the antibody level)

29. 22. Do you consider multi-reactivity for MSA/MAA on blot clinically relevant? *

Mark only one oval.

- Never, independent on clinical information and antibody level
- Clinical relevance is independent of clinical information but dependent on the antibody level
- Clinical relevance is depending on the pre-test probability and/or antibody levels
- No opinion, I refer to lab staff for interpretation
- I will discuss the case with the lab staff to decide on the clinical relevance

30. 22a. Other, please specify:

____
____
____
____
____

31. 23. Some myositis blots contain two antigens for Mi2 (Mi2a and Mi2b). What is your opinion on the clinical relevance of measuring both antigens? (indicate all that apply) *

Mark only one oval.

- ◯ I think both reactivities are equally relevant
- ◯ I think anti-Mi2a is more relevant than anti-Mi2b positivity
- ◯ I think anti-Mi2b is more relevant than anti-Mi2a positivity
- ◯ I think that double positivity is more relevant than single positivity
- ◯ I think that there is no added value of subdifferentiating
- ◯ I don't know
- ◯ My laboratory doesn't report these two specificities independently

32. 24. Some myositis blots contain two antigens for PM/Scl (PM/Scl75 and PM/Scl100). What is your opinion on the clinical relevance of measuring both antigens? (indicate all that apply) *

Mark only one oval.

- I think both reactivities are equally relevant
- I think anti-PM/Scl100 is more relevant than anti-PM/Scl75 positivity
- I think anti-PM/Scl75 is more relevant than anti-PM/Scl100 positivity
- I think that double positivity is more relevant than single positivity
- I think that there is no added value of subdifferentiating
- I don't know
- My laboratory doesn't report these two specificities independently

33. 25. Some myositis blots contain two antigens for SAE (SAE1 and SAE2). What is your opinion on the clinical relevance of measuring both antigens? (indicate all that apply) *

Mark only one oval.

- I think that anti-SAE1 is more relevant than anti-SAE2 positivity
- I think that anti-SAE2 is more relevant than anti-SAE1 positivity
- I think that double positivity is more relevant than single positivity
- I think that there is no added value of subdifferentiating
- I don't know
- My laboratory doesn't report these two specificities independently

34. 26. Anti-Jo1 is usually also part of the standard anti-ENA diagnostics. Do you know how the result of anti-Jo1 antibodies are obtained in y our referent laboratory? *

Mark only one oval.

- Anti-Jo1 is ONLY obtained by standard ENA testing (and not included in the myositis blot)
- Anti-Jo1 is not part of the standard ENA testing and ONLY obtained by the myositis blot
- Anti-Jo1 can be obtained by standard anti-ENA testing as well as myositis blot, and results are combined in an integrated result
- Anti-Jo1 can be obtained by standard anti-ENA testing as well as myositis blot , and results are reported individually
- Anti-Jo1 is ALWAYS tested by all the assays available in the laboratory (standard anti-ENA testing and myositis blot)
- I don't know

35. 27. What is the impact of MSA/MAA testing on your practice? *

Mark only one oval per row.

	Strongly disagree	Disagree	Neither agree nor disagree	Agree	Strongly agree
MSA/MAA testing influences my diagnostic confidence	⬭	⬭	⬭	⬭	⬭
MSA/MAA testing influences the information I provide to a patient on prognosis	⬭	⬭	⬭	⬭	⬭
MSA/MAA testing influences any further investigations I arrange	⬭	⬭	⬭	⬭	⬭
MSA/MAA testing influences my recommended treatment	⬭	⬭	⬭	⬭	⬭

36. 28. In your opinion how useful is the monitoring of the MSA/MAA in the patient follow up ? *

Mark only one oval.

- This is not useful
- It may be interesting to confirm the diagnosis
- It may be interesting to monitor the treatment efficacy
- Otherwise

37. 28a. Otherwise, please specify:

38. 29. Do you repeat testing if unexpectedly negative in context of clinical suspicion? *

Mark only one oval.

- No *Skip to question 41*
- Yes

39. 29 a. If yes, moment of retesting may be influenced by: (indicate all that apply)

Tick all that apply.

- [ ] Clinical changes
- [ ] Treatment resistance
- [ ] Time frame
- [ ] Other: ______________________

40. 29b. Please specify the time frame

____

41. Thank you very much for your participation!
