# Supplementary material for: Myositis-specific and myositis-associated autoantibodies in idiopathic inflammatory myopathies: An EASI survey on current clinical and laboratory practices
Source: J Transl Autoimmun. 2026 Sep 1;13:100398. doi: 10.1016/j.jtauto.2026.100398 (PMC13571532; doi:10.1016/j.jtauto.2026.100398)
Supplement: Supplementary file 2 [file mmc3.pdf]

EASI questionnaire on Myositis Multiplex Assay

Detection of myositis-specific autoantibodies (MSA) and myositis-associated autoantibodies (MAA) are increasingly requested in routine clinical settings.
Detection of these autoantibodies is most often performed by multiplex line or dot immuno-assays (myositis blot). Analysis, results interpretation and reporting appear to be heterogeneous and therefore prone to harmonization. A consensus on harmonization has been reached by the ENMC (Damoiseaux et al, NMD 2022) and this consensus is a lead for this questionnaire.

For this questionnaire the following MSA/MAA are taken into account: anti-Jo1 , -EJ, -OJ, -PL7, -PL1 2, -KS, -ZO, -HA, -SRP, -MDA5, -TIF1y, -SAE, -SAE1 , -SAE2, -NXP2, -Mi2, -Mi2a, -Mi2b, -cN1 A, -HMGCR, -PM/Scl, -PM/Scl-1 00, -PM/Scl-75, -Ku.

The aim of this questionnaire is to understand the variation in laboratory practice, with the ultimate goal to come to a consensus and alignment of the different aspects of the application of the myositis blots.

* Indicates required question

Section 1 - Laboratory description

1. 1. Your laboratory belongs to: *

Mark only one oval.

- An university hospital
- A non-university hospital
- A private hospital
- A private laboratory

2. 1b. Country where your laboratory is located? *

____

3. 2. What is the main specialty of your laboratory ? *

Mark only one oval.

- Immunology
- Biochemistry/Clinical Pathology
- Polyvalent
- Other

4. 3. Do you perform analysis in your lab to identify MSA/MAA? *

Mark only one oval.

- Yes, we perform all analysis in our lab . *Skip to question 5*
- Yes, but we sometimes refer to another lab in case of difficult cases/inconsistent findings . *Skip to question 5*
- Yes, but for some autoantibodies we refer to another lab . *Skip to question 5*
- No, we systematically refer for ALL requests . *Skip to question 7*

Section 2 - numbers of analysis

5. 3a. How many samples do you test per month for myositis blot? *

Mark only one oval.

- 0
- 1 to 10
- 11 to 25
- 26 to 50
- 51 to 100
- >100

6. 3b. How fast do you usually report your MSA/MAA results? *

Mark only one oval.

- 1 -3 day
- 3-7 days
- 8-14 days
- > 14 days

Skip to question 8

Section 3 - numbers of referral

7. 3c. How many samples per month do you refer for the identification of MAA/MSA? *

Mark only one oval.

- 0 *Skip to question 73*
- 1 to 10 *Skip to question 73*
- 11 to 20 *Skip to question 73*
- 21 to 50 *Skip to question 73*
- >50 *Skip to question 73*
- I don't know *Skip to question 73*

Section 4: Laboratory methodology for the identification of MSA (and MAA) (part A)

8. 4. Which type of myositis blot are you using? (multiple answers possible) *

Tick all that apply.

- [ ] Euroimmun 16Ag
- [ ] Euroimmun 16Ag including cN1A
- [ ] Euroimmun 16Ag including HMGCR
- [ ] Euroimmun 16Ag including HMGCR + cN1A
- [ ] Euroimmun 20Ag (including Zo, Ha, Ks)
- [ ] D-tek/ Alphadia Myositis Profile 12 Ag Dot
- [ ] D-tek/ Alphadia Myositis Profile 12 - OJ Ag IgG Dot
- [ ] D-tek/ Alphadia Sclero-Poly-Synthetase Profile 12 Ag Dot
- [ ] D-tek/ Alphadia Sclero-Poly-Synthetase Profile 8 Ag Dot
- [ ] D-tek/ Alphadia Synthetase Profile 10 Ag Dot
- [ ] Other

9. 4a. If you are using 'other' myositis blots than mentioned above, please specify details

Section 5: Laboratory methodology for the identification of MSA (and MAA) (part B)

10. 5. Which reactivities are tested for in your lab using myositis blot(s) (indicate all that apply)? *

Remark: If your blot contains separate antigen dots/lines for the anti-Mi2, -PM/Scl or -SAE subtypes, please indicate them separately (and do not indicate the whole antigen)

Tick all that apply.

- [ ] Mi2
- [ ] Mi2a
- [ ] Mi2b
- [ ] MDA5
- [ ] TIF1y
- [ ] SAE
- [ ] SAE1
- [ ] SAE2
- [ ] NXP2
- [ ] HMGCR
- [ ] SRP
- [ ] JO1
- [ ] PL7
- [ ] PL12
- [ ] EJ
- [ ] OJ
- [ ] ZO
- [ ] HA
- [ ] KS
- [ ] cN1A
- [ ] Ku
- [ ] PM/Scl
- [ ] PM/Scl75

- [ ] PM/Scl100

11 . 5b. Which reactivities are reported for in your lab using myositis blot(s) (indicate all that apply)? *

Tick all that apply.

- [ ] Mi2a, Mi2b or Mi2
- [ ] MDA5
- [ ] TIF1y
- [ ] SAE1, SAE2 or SAE
- [ ] NXP2
- [ ] HMGCR
- [ ] SRP
- [ ] Jo1
- [ ] PL7
- [ ] PL12
- [ ] EJ
- [ ] OJ
- [ ] ZO
- [ ] HA
- [ ] KS
- [ ] cN1A
- [ ] Ku
- [ ] PM/Scl75, PM/Scl100 or PM/Scl

12. 6. For which of the above MSA/MAA do you use alternative immuno-assay(s) other than a myositis blot (also specify the method)?

____

____

____

____

____

13. 7. Do you use an automated preparator for the incubation of the myositis blots? *

Mark only one oval.

- ( ) No, they are incubated manually *Skip to question 15*
- ( ) Yes, EUROBlotOne *Skip to question 15*
- ( ) Yes, EUROBlotMaster *Skip to question 15*
- ( ) Yes, Autoblot 3000 *Skip to question 15*
- ( ) Yes, Blue Diver I *Skip to question 15*
- ( ) Yes, Blue Diver II *Skip to question 15*
- ( ) Yes, with another analyzer or a combination of the above options *Skip to question 14*

Section 6-Details on the type of incubation device(s) used:

14. 7a. If you use another strategy for incubation than mentioned above, please specify:

____

Section 7- Laboratory methodology for the identification of MSA (and MAA) (part C)

15. 8. Do you use a reader system for the analysis of the results? *

Mark only one oval.

- ( ) No, they are read visually *Skip to question 17*
- ( ) Yes, they are read by a reader system *Skip to question 16*

Section 8: Details on the blot reader system used:

16. 8a. Which reader system do you use? *

____

Section 9: Laboratory methodology for the identification of MSA (and MAA) (Part D)

17. 9. How do you report your results? (Indicate all options that apply) *

Tick all that apply.

- [ ] Qualitative (negative or positive) categorized based on the manufacturers cut-off
- [ ] Qualitative (negative or positive) based on an in-house established cut-off
- [ ] Semi-quantitative in scores (e.g.-, +/-, +, ++, ..) categorized using manufacturers cut-off values
- [ ] Semi-quantitative in wording (e.g. weak, moderate, strong positive, ...) categorized using manufacturers cut-off values
- [ ] Semi-quantitative in scores (e.g.-, +/-, +, ++, ..) categorized using in-house established cut-off values
- [ ] Semi-quantitative in wording (e.g. weak, moderate, strong positive, ...) categorized using in-house established cut-off values
- [ ] Quantitative values as generated by the scanner are reported
- [ ] Other

18. 9a. If you use in-house established cut-off values, please specify how it has been done:

____

____

____

____

____

19. 9b. If you report by another way, please specify:

Section 10: Accreditation and quality assurance (part A)

20. 10. Are the different antigen specificities of the myositis blot included in the scope of your ISO 15189 accreditation? *

Mark only one oval per row.

	Included in the scope of our ISO 15189 accreditation	Not included in the scope of our ISO 15189 accreditation	Not present on any of the blots or not performed
Mi2 or Mi2a or Mi2b	◯	◯	◯
MDA5	◯	◯	◯
TIF1y	◯	◯	◯
SAE or SAE1 or SAE2	◯	◯	◯
NXP2	◯	◯	◯
HMGCR	◯	◯	◯
SRP	◯	◯	◯
Jo1	◯	◯	◯
PL7	◯	◯	◯
PL12	◯	◯	◯
EJ	◯	◯	◯
OJ	◯	◯	◯
ZO	◯	◯	◯
HA	◯	◯	◯
KS	◯	◯	◯
cN1A	◯	◯	◯
Ku	◯	◯	◯
PM/Scl or PM/Scl75 or PM/Scl100	◯	◯	◯

21 11. Have you performed a verification* of the distinct antigen specificities? *

*VERIFICATION: is the abbreviated validation process that confirms via objective evidence (by documenting performance characteristics) that an already validated examination procedure (e.g. CE-labelled method such as the lineblot) is appropriate for a specific intended use in one's own laboratory (internal validation).

Mark only one oval per row.

	Yes, verification performed	No verification, but not reported	No verification, relied on kit insert	No verification, relied on publication	No verification, relied on kit insert and publication	Not present on any of the blots
Mi2 or Mi2a or Mi2b	⬭	⬭	⬭	⬭	⬭	⬭
MDA5	⬭	⬭	⬭	⬭	⬭	⬭
TIF1y	⬭	⬭	⬭	⬭	⬭	⬭
SAE or SAE1 or SAE2	⬭	⬭	⬭	⬭	⬭	⬭
NXP2	⬭	⬭	⬭	⬭	⬭	⬭
HMGCR	⬭	⬭	⬭	⬭	⬭	⬭
SRP	⬭	⬭	⬭	⬭	⬭	⬭
Jo1	⬭	⬭	⬭	⬭	⬭	⬭
PL7	⬭	⬭	⬭	⬭	⬭	⬭
PL12	⬭	⬭	⬭	⬭	⬭	⬭
EJ	⬭	⬭	⬭	⬭	⬭	⬭
OJ	⬭	⬭	⬭	⬭	⬭	⬭
ZO	⬭	⬭	⬭	⬭	⬭	⬭
HA	⬭	⬭	⬭	⬭	⬭	⬭
KS	⬭	⬭	⬭	⬭	⬭	⬭
cN1A	⬭	⬭	⬭	⬭	⬭	⬭
Ku	⬭	⬭	⬭	⬭	⬭	⬭
PM/Scl or PM/Scl75 or PM/Scl100	⬭	⬭	⬭	⬭	⬭	⬭

22. 12. How do you manage the report of the specificity(ies) for which you have not performed any verification? *

Mark only one oval.

- I do not report such results
- I report the results with the comment that the performances of this reactivity are not known.
- I report the results with the comment that this reactivity is not part of the IS01 51 89 accreditation.
- All specificities have been verified
- 0ther approach

23. 12a. If you have other approach, could you please specify the management of the reporting of unverified reactivities?

24. 13. Do you participate for the myositis line/dot blot in an EQA/EQC (external quality assurance/control) program? *

Mark only one oval.

- Yes Skip to question 25
- No Skip to question 27

Section 11 : Details on EQA (to be completed for EQA participants only)

25. 13a. Please specify the EQA/EQC program(s): *

____

26. 14. Which antigen-specificities have been covered in the respective EQA/EQC program during the last 3 years (antigens identified as "correct/designated" response" OR present in the majority of answers but classified as "other") ? *

Mark only one oval per row.

	Covered in EQA program as designated response	Present in EQA program as "other antigen" and not as designated answer	Not covered in EQA program	Not relevant, as not tested/reported on the blot
Mi2 or Mi2a or Mi2b				
MDA5				
TIF1y				
SAE or SAE1 or SAE2				
NXP2				
HMGCR				
SRP				
Jo1				
PL7				
PL12				
EJ				
OJ				
ZO				
HA				
KS				
cN1A				
Ku				
PM/Scl or PM/Scl75 or PM/Scl100				

Section 12: IQC management

27. 15. Do you run IQC (meaning that an IQC sample is analyzed together with patient samples) for at least one MAA/MSA? *

Mark only one oval.

- Yes — Skip to question 28
- No — Skip to question 31

Section 13: Details on the IQC management

28. 15a. For which antigen-specificities do you run IQC and with which kind of sample? *

Mark only one oval per row.

	Yes, with a commercial IQC	Yes, with patient 's derived sample(s) (unique or pooled)	Yes, with a commercial IQC	No IQC performed	Not applicable on blot
Mi2 or Mi2a or Mi2b					
MDA5					
TIF1y					
SAE or SAE1 or SAE2					
NXP2					
HMGCR					
SRP					
Jo1					
PL7					
PL12					
EJ					
OJ					
ZO					
HA					
KS					
cN1A					
Ku					
PM/Scl or PM/Scl75 or PM/Scl100					

29. 15b. How frequent do you run an IQC for your myositis blot? *

Mark only one oval.

- Every run
- At lot change only
- Not in every run, but at a fixed frequency, independently of the lot
- change Other approach

30. 15c. If the IQC is used at a fixed frequency, please specify the frequency:

Section 14: Prescription rules and test initiation in the institution

31. 16. Which disciplines can request a myositis blot? *

Mark only one oval.

- all clinical disciplines (including general practitioners)
- all clinical disciplines (except general practitioners)
- only a selected set of clinical disciplines
- the laboratory is not aware about the speciality of physicians who request the tests.

32. 16a. If only a selected set of clinical disciplines can request a myositis blot, please specify:

____
____
____
____
____

33. 17. Is it possible for the clinicians to request a myositis blot independently from other laboratory tests? *

Mark only one oval.

- ( ) Yes
- ( ) No, only in combination with HEp-2 IFA
- ( ) No, only if HEp-2 IFA tested positive

34. 18. Recent ENMC guidelines* state that MSA/MAA should not be requested by general practioners, and that patients* should be referred for the MSA/MMA request to clinical immunologists, rheumatologists, neurologists, dermatologists, or pulmonologists, depending on the dominant clinical manifestation. Do you agree with this proposal?
* https://doi.org/1 0.1 01 6/j.nmd.2022.05.011

Mark only one oval.

- Yes
- No
- I don't know

35. 19. Can the laboratory independently initiate the myositis blot based on HEp-2 IFA pattern (e.g. suspicious cytoplasmic staining) and/or clinical context?

Mark only one oval.

- Yes, but the lab has to inform the clinician
- Yes, the lab can initiate the analysis independently
- No, but the lab can suggest the testing

36. 20. When does the laboratory initiate the identification of MSA/MAA by myositis blot? (indicate all options that apply) *

Tick all that apply.

- [ ] Guided by the request of the clinician
- [ ] Guided by clinical information (independent of the request)
- [ ] Guided by the ANA pattern
- [ ] Other situations

37. 20a. If you initiate the identification of MSA/MAA in other situation(s) that stated above, please specify:

____

38. 21 . Recent ENMC guidelines* listed clinical indications that ask for the initiation of a MSA/MAA analysis. Do you agree with this list of indications?*

* https://doi.org/10.1016/j.nmd.2022.05.011

Table 1A
Myositis syndrome features that ask for detection of myositis specific autoantibodies.

The triad of myositis, interstitial lung disease, and arthritis, possibly accompanied by Raynaud's phenomenon, mechanic's hands, and fever
Characteristic skin rash, including Gottron's papules, shawl sign, and heliotrope rash, most often in combination with symmetrical proximal muscle weakness
Severe proximal muscle weakness with a sub-acute onset and without clinical extra-muscular manifestations
Slowly progressive muscle weakness with an asymmetrical distribution involving both proximal and distal muscles, typically presenting after the age of 40 (only anti-cN1A*)

*Formally, anti-cN1A is considered not to be a myositis specific autoantibody.

Table 1B
Myositis syndrome features for which detection of myositis specific autoantibodies should be considered after excluding other, more common, diagnoses.

Isolated seronegative and non-erosive polyarthritis*
Interstitial Lung Disease of unknown cause
Isolated high CK level on repeated samples
LGMD-like disease with no known molecular diagnosis nor familial history**

*For interpretation the focus should be only on the anti-synthetase antibodies. Anti-Jo1 detection, most often, is already included in the anti-ENA analysis, but may go unnoticed in case of negativeHEp-2 IFA results.
**For interpretation the focus should be only on the anti-SRP and anti-HMGCR antibodies.

Mark only one oval.

- ◯ Yes
- ◯ No

39. 21a. If you do not agree with the indication list: please specify your concerns:

____
____
____
____
____

Section 15: Laboratory approach (general part)

40. 22. Are you using (or: do you consider appropriate?) ANA/HEp-2 IFA for screening for MSA/MAA? *

Mark only one oval.

- Yes
- No

41. 22a. Please justify your answer:

____
____
____
____
____

42. 23. Do you use HEp-2 IFA patterns (or do you consider them appropriate) to 'support' MSA/MAA findings if the pattern is compatible?

Mark only one oval.

- Yes
- No

43. 23a. According to your opinion, for what MSA correlating the (positive) blot results with the HEp-2 IFA patterns is of added value?

44. 23b. Do you apply a minimal time frame for retesting? *

Mark only one oval.

- No
- Yes

45. 23c. If yes, specify...

____

46. 24. Do you use the Euroimmun blot for the identification of MSA/MAA? *

Mark only one oval.

- ( ) Yes Skip to question 47
- ( ) No Skip to question 51

Section 16: Laboratory_approach — Euroimmun users reporting

47. 25a. Do you apply semi-quantitive or qualitative reporting of the Euroimmun results (independent of whether you also report the quantitative values)?*

Additional information:
- Qualitative reporting (positive/negative) without providing further information on the level of the antibody signal

- Semi-quantitative reporting with grading depending on the antibody level either in symbols (e.g. +, ++,+++) or in words (e.g. weak positive, moderate positive, strong positive, etc.)

Mark only one oval.

- ( ) Yes Skip to question 48
- ( ) No Skip to question 51

Section 17: Laboratory_approach — Euroimmun users reporting - details

48. 25. What do you report as weak positive results from the Euroimmun reader system? *

Mark only one oval.

- 6-10 U (8-14 U for Euroblot one users)
- 11-25 U (15-35 U for Euroblot one users)
- Other (eg. In house cut-off values)

49. 25a. Do you apply the same reporting strategy for weak positive results for all MSA/MAA? *

Mark only one oval.

- Yes
- No

50. 25b. If no, please specify...

____

Section 18: Laboratory_approach — D-tek/Alphadia users

51. 27a. Do you use the D-tek/Alphadia blot for the identification of MSA/MAA? *

Mark only one oval.

- Yes Skip to question 52
- No Skip to question 56

Section 19: Laboratory_approach — D-tek/Alphadia users- details (part 1)

52. 27b. Do you apply semi-quantitative or qualitative reporting of the D-tek results (independent of whether you also report the quantitive values)? *

Additional information:
- Qualitative reporting (positive/negative) =without providing further information on the level of the antibody signal

- Semi-quantitative reporting =with grading depending on the antibody level either in symbols (e.g. +, ++,+++) or in words (e.g. weak positive, moderate positive, strong positive, etc.)

Mark only one oval.

- Yes Skip to question 53
- No Skip to question 56

Section 20: Laboratory_approach — D-tek/Alphadia users- details (part 2)

53. 27c. What do you report as weak positive results from the D-tek/Alphadia reader system? *

Mark only one oval.

- We do not report weak positive results
- 5-10 U
- Other

54. 27d. Do you apply the same reporting strategy for weak positive results for all MSA/MAA? *

Mark only one oval.

- No
- Yes

55. 27e. If no, please specify...

Section 21: Laboratory approach — Combination of assays and results

56. 28. Are positive results obtained in the first-line myositis blot confirmed on an alternative antigen-specific immuno-assay (e.g. a second line/dot blot and/or other methodology, but not HEp-2 IFA) further referred to as ‘the confirmation assay’? (indicate all that apply) *

Tick all that apply.

	No, no other assay available	Yes but this confirmation needs a specific request from the clinician	Yes, by performing a different myositis blot	Yes, by performing another immunoassay	Yes, but only in specific situations (e.g. discrepancy between IFA and blot, low titers, inconsistent clinical findings , ...	Not applicable because not present on any of the blots performed
Mi2	☐	☐	☐	☐	☐	☐
Mi2a	☐	☐	☐	☐	☐	☐
Mi2b	☐	☐	☐	☐	☐	☐
MDA5	☐	☐	☐	☐	☐	☐
TIF1y	☐	☐	☐	☐	☐	☐
SAE	☐	☐	☐	☐	☐	☐
SAE1	☐	☐	☐	☐	☐	☐
SAE2	☐	☐	☐	☐	☐	☐
NXP2	☐	☐	☐	☐	☐	☐
HMGCR	☐	☐	☐	☐	☐	☐
SRP	☐	☐	☐	☐	☐	☐
Jo1	☐	☐	☐	☐	☐	☐
PL7	☐	☐	☐	☐	☐	☐
PL12	☐	☐	☐	☐	☐	☐
EJ	☐	☐	☐	☐	☐	☐
OJ	☐	☐	☐	☐	☐	☐
ZO	☐	☐	☐	☐	☐	☐
HA	☐	☐	☐	☐	☐	☐
KS	☐	☐	☐	☐	☐	☐
cN1A	☐	☐	☐	☐	☐	☐
Ku	☐	☐	☐	☐	☐	☐
PM/Scl	☐	☐	☐	☐	☐	☐
PM/Scl75	☐	☐	☐	☐	☐	☐
PM/Scl100	☐	☐	☐	☐	☐	☐

57. 28a. If you performed another specific immunoassay, please specify:

58. 29. If you perform at least one confirmation assay, how do you manage the discordant results? *

Mark only one oval.

- I report all the individual results of the different tests and let the clinician conclude
- I report all the individual results of the different tests and comment (e.g. on clinical point of view and assay-dependent variability, antigen presentation)
- I integrate the individual results and report one combined result interpretation
- I do not perform confirmation assay

59. 30. How do you report <u>multi-reactivity for MSA/MAA</u> results as obtained on myositis blot? (indicate all that apply) *

Tick all that apply.

- [ ] All MSA/MAA blot results are reported as they are, without any comment
- [ ] All MSA/MAA blot results are reported as they are, but a comment is added
- [ ] In case of multi-reactivity, for all MSA/MAA, weak positive results are reported negative
- [ ] In case of multi-reactivity, for some MSA/MAA, weak positive results are reported negative
- [ ] Multiple reactivities on blot are elaborated with a confirmation assay and only a combined result interpretation is provided
- [ ] Multiple reactivities on blot are elaborated with a confirmation assay and the individual results of the different tests are reported with a comment

60. 30b. If you add a comment, please specify:

____

____

____

____

____

61. 30c. In your opinion, how useful is the monitoring of the MSA/MAA in the patient follow up?*

Mark only one oval.

- This is not useful
- It may be interesting to confirm the diagnosis
- It may be interesting to monitor the treatment efficacy
- Otherwise

62. 30d. Otherwise, please specify

____

Section 22: Reporting and clinical interpretation of some specific antigens

63. 31. Some myositis blots contain two antigens for Mi2 (Mi2a and Mi2b). How do you report these results?*

Mark only one oval.

- ( ) Our myositis blot does not contain this combination
- ( ) We report as independent specificities
- ( ) We report only the result of anti-Mi2a
- ( ) We report only the result of anti- Mi2b
- ( ) We report as anti-Mi2 positive only if both entities are positive
- ( ) We report as anti-Mi2 positive if at least one of both entities is positive
- ( ) Otherwise

64. 31a. If you apply another strategy for anti-Mi2 than listed above, please specify.

____
____
____
____
____

65. 31b. Some myositis blots contain two antigens for Mi2 (Mi2a and Mi2b). What is your opinion on the clinical relevance of measuring both antigens?*

Mark only one oval.

- I think both reactivities are equally relevant
- I think anti-Mi2b is more relevant than anti-Mi2a
- I think anti-Mi2a is more relevant than anti-Mi2b
- I think double positivity is more relevant than single positivity
- I think there is no added value of subdifferentiating
- I do not know

66. 32. Some myositis blots contains two antigens for PM/Scl (PM/Scl75 and PM/Scl1 00). How do you report these results? *

Mark only one oval.

- Our myositis blot does not contain this combination
- We report as independent specificities
- We report only the result of anti-PM/Scl75
- We report only the result of anti-PM/Scl100
- We report as anti-PM/Scl positive if both entities are positive
- We report as anti-PM/Scl positive if at least one of both entities is positive
- Otherwise
- Other:

67. 32a. If you apply another strategy for anti-PM/Scl than listed above, please specify.

68. 32b. Some myositis blots contain two antigens for PM/Scl (PM/Scl75 and PM/Scl1 00). What is your opinion on the clinical relevance of measuring both antigens?*

Mark only one oval.

- I think both reactivities are equally relevant
- I think anti-PM/Scl100 is more relevant than anti-PM/Scl75
- I think anti-PM/Scl75 is more relevant than anti-PM/Scl100
- I think double positivity is more relevant than single positivity
- I think there is no added value of subdifferentiating
- I do not know

69. 33. Some myositis blots contain two antigens for SAE (SAE1 and SAE2). How do you report these results? *

Mark only one oval.

- Our myositis blot does not contain this combination
- We report as independent specificities
- We report only the result of anti-SAE1
- We report only the result of anti-SAE2
- We report as anti-SAE positive if both entities are positive
- We report as anti-SAE positive if at least one of both entities is positive
- Otherwise

70. 33a. If you apply another strategy for anti-SAE than listed above, please specify.

71. 33b. Some myositis blots contain two antigens for SAE (SAE1 and SAE2). What is your opinion on the clinical relevance of measuring both antigens?*

Mark only one oval.

- I think both reactivities are equally relevant
- I think anti-SAE1 is more relevant than anti-SAE2
- I think anti-SAE2 is more relevant than anti-SAE1
- I think double positivity is more relevant than single positivity
- I think there is no added value of subdifferentiating
- I do not know

72. 34. Anti-Jo1 is usually also part of the standard anti-ENA diagnostics. How the result of anti-Jo1 antibodies are obtained in your laboratory?*

Mark only one oval.

- Anti-Jo1 is ONLY obtained by standard ENA testing (and not included in the myositis blot)
- Anti-Jo1 is not part of the standard ENA testing and ONLY obtained by the myositis blot
- Anti-Jo1 can be obtained by standard anti-ENA testing as well as myositis blot, and results are combined in an integrated result
- Anti-Jo1 can be obtained by standard anti-ENA testing as well as myositis blot, and results are reported individually
- Anti-Jo1 is ALWAYS tested by all the assays available in the laboratory (standard anti-ENA testing and myositis blot)

73. Thank you very much for your participation!
