# Supplementary material for: Myositis-specific and myositis-associated autoantibodies in idiopathic inflammatory myopathies: An EASI survey on current clinical and laboratory practices
Source: J Transl Autoimmun. 2026 Sep 1;13:100398. doi: 10.1016/j.jtauto.2026.100398 (PMC13571532; doi:10.1016/j.jtauto.2026.100398)
Supplement: Supplementary file 3 [file mmc1.docx]

**Supplementary materials**

- Questions clinicians (separate file)
- Questions laboratory medicine specialists (separate file)
- Supplemental Table 1.

**Supplemental Table 1.** Geographical distribution of laboratory specialist that participated in the questionnaire.

| **Country** | **Number of laboratory specialist participants  n (%)** |
| --- | --- |
| Australia | 1 (1%) |
| Austria | 3 (3%) |
| Belgium | 4 (4%) |
| Canada | 1 (1%) |
| Croatia | 2 (2%) |
| Czech Republic | 2 (2%) |
| Denmark | 2 (2%) |
| Estonia | 1 (1%) |
| France | 17 (17%) |
| Germany | 2 (2%) |
| Hungary | 4 (4%) |
| Ireland | 2 (2%) |
| Italy | 7 (7%) |
| Lithuania | 1 (1%) |
| Netherlands | 11 (11%) |
| New Zealand | 1 (1%) |
| Portugal | 6 (6%) |
| Slovenia | 1 (1%) |
| South Africa | 1 (1%) |
| Spain | 15 (15%) |
| Sweden | 2 (2%) |
| Switzerland | 4 (4%) |
| United Kingdom | 10 (10%) |
| **Total** | **100** |
